# Variant calling enhances the identification of cancer cells in single-cell RNA sequencing data

**DOI:** 10.1371/journal.pcbi.1010576

**Published:** 2022-10-03

**Authors:** William Gasper, Francesca Rossi, Matteo Ligorio, Dario Ghersi

**Affiliations:** 1 School of Interdisciplinary Informatics, University of Nebraska at Omaha, Omaha, Nebraska, United States of America; 2 Department of Surgery, University of Texas Southwestern, Dallas, Texas, United States of America; University College London, UNITED KINGDOM

## Abstract

Single-cell RNA-sequencing is an invaluable research tool that allows for the investigation of gene expression in heterogeneous cancer cell populations in ways that bulk RNA-seq cannot. However, normal (i.e., non tumor) cells in cancer samples have the potential to confound the downstream analysis of single-cell RNA-seq data. Existing methods for identifying cancer and normal cells include copy number variation inference, marker-gene expression analysis, and expression-based clustering. This work aims to extend the existing approaches for identifying cancer cells in single-cell RNA-seq samples by incorporating variant calling and the identification of putative driver alterations. We found that putative driver alterations can be detected in single-cell RNA-seq data obtained with full-length transcript technologies and noticed that a subset of cells in tumor samples are enriched for putative driver alterations as compared to normal cells. Furthermore, we show that the number of putative driver alterations and inferred copy number variation are not correlated in all samples. Taken together, our findings suggest that augmenting existing cancer-cell filtering methods with variant calling and analysis can increase the number of tumor cells that can be confidently included in downstream analyses of single-cell full-length transcript RNA-seq datasets.

## Author summary

Tumor samples in single-cell RNA sequencing datasets often contain non cancerous cells that may confound downstream analyses. Therefore, an important first step before performing further analyses is to filter the data and enrich for cancer cells. A common approach to enrich for cancer cells in single-cell sequencing datasets is to look for high copy number variation. However, not all cancer types and not all cells within a cancer are equally prone to high copy number variation. In this work, we propose to augment the filtering process by incorporating variant calling and putative driver alterations. We analyzed a triple negative breast cancer and a colorectal cancer dataset, and showed that driver alterations can be identified in cells that have low copy number variation. Further, gene expression in these cells is highly enriched for classic tumor pathways, suggesting that these cells are likely cancerous. Overall, our work adds another important dimension to the analysis of transcriptomics data at the single-cell level.

## Introduction

Single-cell transcriptomics is revolutionizing the way we look at cancer. The ability to measure gene expression at the single cell level is particularly important given the high degree of intratumor heterogeneity exhibited by human cancers [1]. Intratumor heterogeneity—often promoted by genomic instability—leads to the emergence of different cancer cell subclones, some of them promoting metastasis, drug resistance, and disease recurrence [2]. The molecular characterization of cancer subclones via single-cell transcriptomics is therefore essential for achieving more effective, personalized treatment options and for improving clinical outcomes.

A fundamental prerequisite for an effective downstream analysis of single-cell transcriptomics datasets is the correct identification of cancer cells, which is often complicated by low tumor purity (i.e., low proportion of tumor cells in cancer samples). In addition to low tumor purity, the cellular process known as epithelial-to-mesenchymal transition (EMT) can further complicate the identification of tumor cells [3]. EMT may cause epithelial cells to express fibroblast-like transcriptional programs and may make (*i*) cancer-associated fibroblasts virtually indistinguishable from cancer cells undergoing EMT, or (*ii*) non-EMT cancer cells indistinguishable from the normal epithelial cells in the surrounding organs (e.g., breast, prostate, pancreas) [3].

Bioinformatics methods are used to selectively call cancer cells in single-cell transcriptomics datasets. Some of these methods rely on the analysis of marker gene expression to identify normal cell types [4] or perform gene expression-based clustering [5]. While these methods add valuable information to the cell filtering process, they leave the potential for both false positives and false negatives and may not be ideal for distinguishing cancer cells from normal epithelial cells, especially in the phenotype discovery phase. In addition, the EMT phenomenon mentioned above can reduce the effectiveness of gene expression-based approaches.

Other *in silico* tools infer tumor cell specific genetic variants, such as copy number variation (CNV), from single-cell RNA-seq (scRNA-seq) data. CNV is defined by duplications or deletions of chromosome parts and is considered a cancer-specific signature often associated with clinical outcomes [6]. One example of a popular CNV-based tool is InferCNV, which was initially developed to infer large-scale CNV for glioblastoma cells with transcriptomics data [7]. Other tools include HoneyBADGER [8], CaSpER [9], and CopyKAT, which identifies CNVs at a resolution of 5 Mb and is suitable for both Smart-seq2 and 10X Genomics-generated scRNA-seq data [10].

CNV-based tools have proven to be effective at calling cancer cells for a variety of malignancies. However, CNV inference based filtering has limited efficacy when applied to samples from certain cancers commonly characterized by limited CNV. Examples of low-CNV cancers include subsets of prostate cancers [11], thyroid carcinomas, and clear cell renal carcinomas [12]. Even in tumor types commonly characterized by medium-to-high CNV, these methods would falsely exclude tumor subclones with low CNV, which we have identified in this work.

To address these issues, we propose variant calling analysis as an additional method to aid in identifying cancer cells in transcriptomics data. Our approach focuses on inferring coding sequence alterations that are known to be genetic drivers in tumor initiation and progression. Variant calling and analysis provides valuable information when attempting to identify cancer cells, particularly in low-CNV tumors, or potentially targetable driver mutations in scRNA-seq data. Our presented methodology and findings suggest that the analysis of variants identified in scRNA-seq data obtained with full-length transcript technologies is underutilized and may yield novel biological insights. This added context will allow us to better understand the mechanisms of tumor progression with the final goal of improving the clinical outcomes for cancer patients.

## Results

Given that not all cancer cells exhibit high levels of CNV, we set out to improve the identification of cancer cells in single-cell full-length transcript RNA sequencing data by combining CNV inference with variant calling. Our novel method removes common SNPs and selects variants that are annotated as known or predicted drivers in OncoKB [13]. Considering that: (1) single-cell RNA datasets do not always have germline samples to compare against; and (2) normal cells can also harbor variants (a few of which can potentially be putative drivers), our method compares the driver variant load in tumor samples with that found in normal samples. Comparison with single-cell normal samples allows our approach to identify cells with a much higher driver load than expected in normal, thereby inferring possible cancer status.

### CNV inference

**Fig 1:**
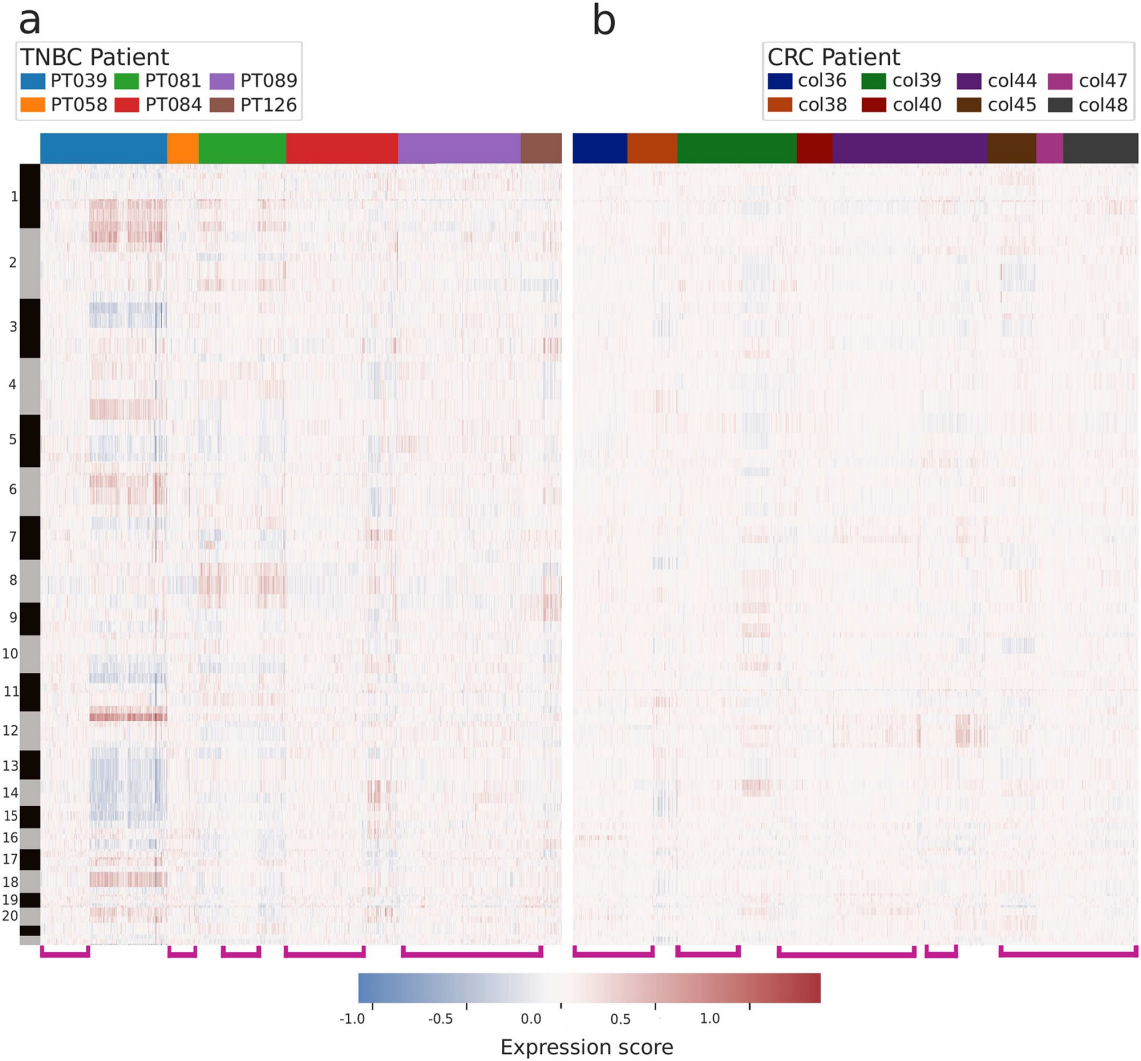
Heatmap visualizing inferred CNV results obtained using CopyKAT [10]. Columns represent individual cells, rows represent genes arranged by chromosomal position. Alternating bars on the y-axis indicate chromosome. Cells are clustered hierarchically within each patient using expression score matrices. Pink brackets indicate cells with low inferred CNV. Panel **(A)** shows results for the TNBC dataset and panel **(B)** for the CRC dataset.

We performed CNV inference using CopyKAT [10] to assess the viability of cancer cell calling based solely on CNV inference. CopyKAT identified structural genomic heterogeneity both within and between patients for both cancer datasets. In the TNBC (triple negative breast cancer) dataset, a significant number of cells had limited or no CNV, and CopyKAT predicted 135 out of 942 cells as aneuploid. For the cells predicted as aneuploid, all but one (belonging to PT081) belonged to PT039. These predicted aneuploid cells are clearly evident in Fig 1a as the cluster of PT039 cells with comparatively extreme expression scores. For the CRC (colorectal cancer) dataset, CopyKAT predicted 227 cells as aneuploid, but this is derived from automatic partitioning based on the lowest inferred CNV cells, and the majority of the cells in the CRC dataset showed limited inferred CNV (Fig 1b).

The presence of large, contiguous regions of high or low expression scores may indicate major structural alterations: either aneuploidy or considerable copy number alteration. Groups of cells predicted to have limited or no structural alterations are highlighted by pink brackets along the x-axis of Fig 1. Interestingly, all cells for patient PT058 had low CNV scores (Fig 1a), with a mean of 0.08, and would likely be filtered out based on the CNV criterion alone. Similarly, the vast majority of cells in the CRC dataset appear to have limited inferred CNV. These results provide the rationale for including additional criteria like the presence of putative driver mutations.

### Variant calling and annotation

**Fig 2:**
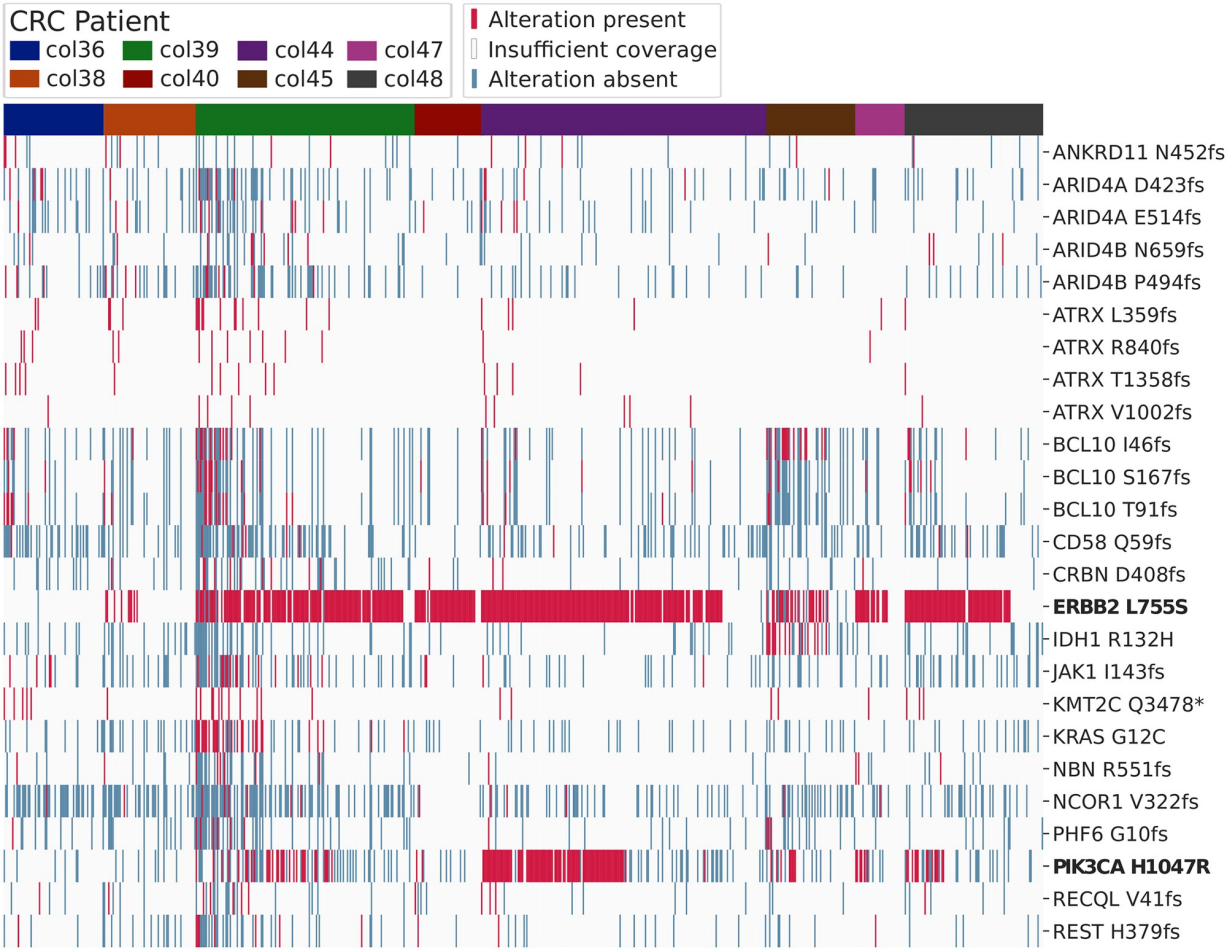
Heatmap indicating alteration status for 736 cells for the top 25 most frequent oncogenic, predicted oncogenic, and likely oncogenic alterations in the CRC dataset. Alterations are annotated using OncoKB. Absence of an alteration is noted when a cell has a read depth of at least 5 for all bases corresponding to the residue. For residues without an oncogenic alteration and with read depths less than 5 for all corresponding bases, the presence or absence of an alteration is not characterized (labeled as “Insufficient coverage”). Common recurrent driver mutations are shown in bold.

In order to investigate the possibility that cells predicted to have limited structural alterations are indeed cancer cells, we examined whether they harbored putative driver alterations. Variant calling on both the CRC and TNBC datasets resulted in the identification of a number of putative driver alterations, defined as those alterations annotated by OncoKB as “Oncogenic”, “Likely Oncogenic”, or “Predicted Oncogenic”. These results are visualized for the 25 most frequent putative driver alterations for the CRC dataset in Fig 2.

For the CRC dataset, a substantial number of cells harbored known, recurrent driver alterations. Most commonly, cells harbored the ERBB2 L755S and PIK3CA H1047R alterations. We found the ERBB2 L755S alteration in 454 cells, the PIK3CA H1047R alteration in 151 cells, and the combination of both in 147 cells. Additionally, 88 cells with the ERBB2 L755S had reasonable coverage for bases in the PIK3CA 1047 residue and are predicted to lack a PIK3CA H1047R mutation, hinting at the potential presence of subclones. Both ERBB2 and PIK3CA are frequently studied oncogenes [14, 15], and the ERBB2 L755S and PIK3CA H1047R alterations have specifically been characterized as recurrent hotspots [16]. The ERBB2 L755S alteration has been implicated in HER2-targeted therapy resistance in breast cancer [17, 18]. HER2-targeting therapies, like trastuzumab and lapatinib, are not typically used in CRC treatments, however these treatments have been investigated for use with CRC [19, 20], and results from breast cancer studies regarding the L755S alteration may be validated in CRC to help develop treatment regimens. The PIK3CA H1047R alteration has been implicated in therapy resistance with CRC, specifically regarding a folinic acid, fluorouracil, and oxaliplatin (FOLFOX) treatment, which is frequently used in clinical practice [21].

In the TNBC dataset, we found several examples of characteristic mutations common to multiple cells: for PT039, the TP53 Y205C (found in 41 cells), TP53 P72R (27), and NCOR1 V322fs (14) alterations; and for PT089, the KMT2C Q3478* (6). PT039 had a number of cells (24) containing multiple TP53 mutations, which seems to be a characteristic signature for these cells within this patient’s sample. There were also 121 alterations common to more than one and less than six cells per patient, and then a large number (1461) of singleton mutations occurring in only one cell per patient. These rare mutations are called based on high quality scores (≥ 30) and may represent genuine genomic variation, but should not be expected to be confirmed in WES or whole-genome sequencing, both of which capture genomic variation common to large numbers of cells. A visualization of the top 25 most frequent putative driver alterations for the TNBC dataset is shown in S1 Fig.

A number of cells (59 in the CRC dataset, 461 in the TNBC dataset) harbored no putative driver alterations. For TNBC dataset cells, a total of 1,448 unique putative driver alterations were identified with a median of 1.0 per cell. In terms of all variants, including non-coding-sequence variants, at least one variant was identified in every cell, with a median of 1,171 variants per cell in the TNBC dataset. For the CRC dataset, 1,101 unique putative driver alterations were found with a median of 3.0 per cell, and, when including non-coding-sequence variants, a median of 7,286 were found per cell.

Additionally, we quantified the presence of splice site alterations in single cells and found that the CRC dataset was significantly enriched in splice site alteration counts versus normal cells. For this analysis, we examined the number of alterations annotated by SnpEff as splice donor, splice acceptor, or splice region variants. A kernel density plot showing the distributions of splice site variant counts for cells is shown in S2 Fig. Although both the CRC and TNBC datasets were significantly different from the normal cell distribution (Mann-Whitney U; *p* < 9.75 ⋅ 10^−230^ and *p* < 0.0016, for CRC and TNBC, respectively), the effect size for TNBC versus normal was negligible (Cliff’s delta; *δ* = 0.064).

These results indicate that variant calling on scRNA-seq data with sufficient coverage can add complementary information into the cell filtering process. Variant analysis may be useful when inferred CNV provides limited evidence towards a positive cancer status for a cell. For example, the entire CRC dataset shows limited inferred CNV (Fig 1), despite having predicted recurrent driver alterations in 458 cells (Fig 2). These findings suggest that actionable information can be added through variant calling and annotation. The addition of this data can allow for the inclusion of additional true cancer cells that might otherwise be excluded.

### Normal tissue analysis

We also investigated the presence of predicted oncogenic driver alterations in scRNA-seq data generated from normal, healthy patients for neutrophils (obtained from saliva), pancreas, dermal fibroblast, and liver cells in order to ensure that our putative driver alteration counts represented a cancer dataset specific phenomenon. Limited numbers of putative driver alterations were found in normal tissue scRNA-seq data: a plurality of healthy cells (51.3%) contained 0 putative oncogenic alterations, with an absolute maximum of 9 putative driver alterations, a mean of 0.92, and a median of 0.0. Putative driver alteration counts were found to be significantly different for both CRC and TNBC datasets when compared to normal cells (Mann-Whitney U; *p* < 3.14 ⋅ 10^−187^ and *p* < 0.013 for CRC and TNBC, respectively). Box plots comparing the normal cell putative driver alteration counts to TNBC and CRC patients are shown in S3 Fig. For CNV inference, the mean CNV expression score for normal tissue cells was 0.06 with an absolute maximum of 0.14. In comparison, the means for the CRC and TNBC datasets were 0.05 and 0.09, respectively, and the distributions were found to be significantly different (Mann-Whitney U; *p* < 5.89 ⋅ 10^−235^ and *p* < 1.79 ⋅ 10^−29^). Box plots visualizing these distributions are shown in S4 Fig. Based on these results, we do not expect that cells with a known normal status outside of cancer datasets should contain the high numbers of predicted driver alterations or the high mean absolute CNV expression scores present in a number of the CRC and TNBC dataset cells.

### Relationship to inferred CNV

**Fig 3:**
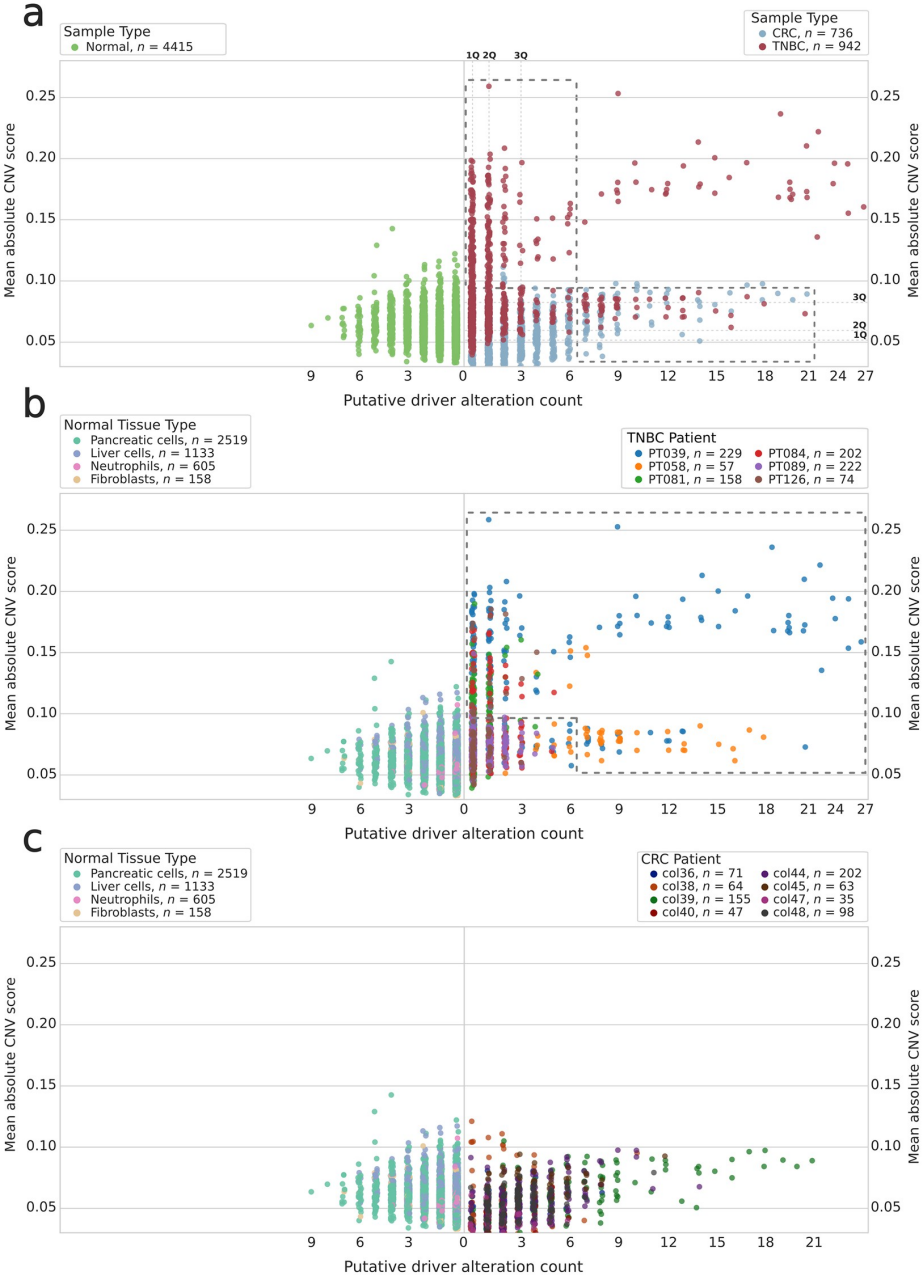
Relationship between putative driver alteration counts and inferred CNV for normal tissues (left) and tumor (right) dataset cells. **(A)** Cancer dataset (at right) cells shown based on primary tumor site, normal cells shown together at left. **(B)** TNBC cells, grouped by patient, shown in comparison to normal cells, grouped by tissue type. **(C)** CRC cells, grouped by patient, shown in comparison to normal cells, grouped by tissue type. Higher mean absolute CNV values indicate predicted structural alterations resulting in copy number variation, and lower values suggest limited CNV. Dashed rectangles in (A) indicate regions of interest: groups of cells that might be identified as cancer cells by either CNV inference or putative driver alteration count. Dashed rectangles are bounded at the lower ends by the 99th percentile values derived from the values for 4,415 normal cells amenable to both CopyKAT and variant analysis. Dashed polygon in (B) indicates cells of interest, with either high CNV scores or high putative driver counts, that might be selected for downstream analyses. In (A), first, second, and third quartiles are indicated for tumor cells by dashed lines and bold labels along their respective axes.

Having established that putative driver alterations are rare or absent in normal cells, we set out to determine whether including putative driver alterations can effectively augment the number of predicted cancer cells when compared to CNV inference alone. In order to do this, we studied the relationship between putative driver alterations and inferred CNV in tumor samples (Fig 3).

A number of cells with limited inferred CNV had high numbers of putative driver alterations. For example, 125 cells from the CRC and TNBC datasets are below the 99th percentile of normal tissue mean absolute CNV scores but above the 99th percentile of normal tissue putative driver alteration counts. Some of these cells of interest are indicated in Fig 3A by the lower dashed rectangle. This represents cells that might be excluded by CNV-inference-based filtering but are likely to be tumor cells. For the majority of CRC samples, these predicted low-CNV cells represent the bulk of the data.

Overall, there is low correlation between the putative driver alteration counts and mean absolute CNV expression score. However, this varies widely based on patient: PT058, PT084, PT089, col38, col40, and col47’s cells had no correlation between CNV and alterations, and the relationship is weak, albeit significant, for PT081 (Table 1).

**Table 1:** Table showing the correlation coefficients and statistical significance for relationships between inferred CNV and putative driver alteration counts for cells belonging to each patient. p-values less than 0.05 are shown in bold.

Patient	Pearson’s *r*	p-value	Spearman’s *r*	p-value
PT039	0.361	**1.80 ⋅ 10^−8^**	0.306	**2.34 ⋅ 10^−6^**
PT126	0.308	**0.008**	0.293	**0.011**
PT081	0.128	0.109	0.122	0.127
PT058	-0.077	0.57	0.099	0.465
PT084	0.014	0.839	0.039	0.584
PT089	0.025	0.716	0.034	0.615
col39	0.604	**8.29 ⋅ 10^−17^**	0.592	**5.22 ⋅ 10^−16^**
col44	0.486	**2.39 ⋅ 10^−13^**	0.437	**7.74 ⋅ 10^−11^**
col45	0.543	**4.21 ⋅ 10^−6^**	0.489	**4.82 ⋅ 10^−5^**
col36	0.302	0.**011**	0.374	**0.001**
col48	0.28	**0.005**	0.22	**0.03**
col38	0.153	0.225	0.161	0.201
col40	0.249	0.092	0.151	0.31
col47	0.059	0.738	0.162	0.354

In the context of CNV inference, the addition of putative driver alteration counts represents an additional dimension that can be effectively used to separate cells. Cells that have similar predicted degrees of structural alteration may be on opposite ends of the alteration counts distribution, and this added dimension allows for the separation of cells that appear similar solely in the context of inferred CNV.

### Gene set enrichment analysis

Accumulation of putative drivers, or the presence of specific drivers, may indicate phenotypic heterogeneity, and we investigated this through gene set enrichment analysis (GSEA) [22]. In order to assess the potential for enrichment in cancer-specific pathways, we analyzed groups of cells for enrichment in MSigDB hallmark gene sets [23], many of which correspond to cancer phenotypes. We compared cells grouped based on the number of drivers identified for CRC and TNBC datasets, and groups of cells corresponding to ERBB2 L755S/PIK3CA H1047R alteration status (positive for either or negative for both) for the CRC dataset. When examining cells in high versus low driver counts groups, we utilized a cutoff of 5 putative drivers (high, > 5 putative drivers; low, ≤ 5 putative drivers), corresponding to the 99th percentile value found in the normal cell dataset. Enrichment results for high driver and driver positive groups versus all others are shown in Table 2.

**Table 2:** Gene set enrichment analysis results showing top 10 enriched cancer hallmark gene sets by enrichment score for groups of driver enriched cells versus all others. Top section shows enrichment when comparing cells with high putative driver counts to cells with low putative driver counts for the CRC dataset. Middle section shows enrichment when comparing ERBB2+/PIK3CA+ cells to cells lacking the characteristic ERBB2 or PIK3CA mutations for the CRC dataset. Bottom section shows enrichment when comparing cells with high putative driver counts to cells with low putative driver counts for the TNBC dataset.

Comparison	Group	Gene set	Enrichment score	FDR q-value
CRC cells with high (> 5, *n* = 119) putative drivers versus low (≤ 5, *n* = 617)	High drivers	Hallmark MYC Targets V1	0.62	0.0
		Hallmark MYC Targets V2	0.57	0.0
		Hallmark Oxidative Phosphorylation	0.57	0.006
		Hallmark E2F Targets	0.53	0.0
		Hallmark G2M Checkpoint	0.49	0.0
		Hallmark Peroxisome	0.46	0.0
		Hallmark PI3K AKT MTOR Signaling	0.46	0.008
		Hallmark Fatty Acid Metabolism	0.46	0.014
		Hallmark Protein Secretion	0.45	0.126
		Hallmark MTORC1 Signaling	0.44	0.015
	Low drivers	Hallmark IL6 JAK STAT3 Signaling	-0.18	0.874
		Hallmark Inflammatory Response	-0.23	0.537
CRC driver positive (ERBB2 L755S or PIK3CA H1047R, *n* = 458) cells versus driver negative cells (*n* = 278)	Driver positive	Hallmark MYC Targets V1	0.51	0.0
		Hallmark Allograft Rejection	0.5	0.0
		Hallmark TGF Beta Signaling	0.49	0.0
		Hallmark Pancreas Beta Cells	0.48	0.157
		Hallmark Interferon Alpha Response	0.45	0.002
		Hallmark PI3K AKT MTOR Signaling	0.45	0.002
		Hallmark Reactive Oxygen Species Pathway	0.43	0.074
		Hallmark Interferon Gamma Response	0.43	0.004
		Hallmark WNT Beta Catenin Signaling	0.42	0.105
		Hallmark Apoptosis	0.42	0.004
	Driver negative	Hallmark KRAS Signaling Down	-0.18	0.862
		Hallmark Bile Acid Metabolism	-0.21	0.614
		Hallmark Coagulation	-0.23	0.588
TNBC cells with high (> 5, *n* = 103) putative drivers versus low (*n* = 839)	High drivers	Hallmark WNT Beta Catenin Signaling	0.58	0.0
		Hallmark TGF Beta Signaling	0.54	0.0
		Hallmark Hedgehog Signaling	0.52	0.015
		Hallmark Notch Signaling	0.51	0.005
		Hallmark IL6 JAK STAT3 Signaling	0.5	0.0
		Hallmark Mitotic Spindle	0.49	0.0
		Hallmark Hypoxia	0.47	0.0
		Hallmark UV Response Up	0.45	0.003
		Hallmark TNFA Signaling via NFKB	0.44	0.019
		Hallmark Apical Junction	0.42	0.003
	Low drivers	Hallmark E2F Targets	-0.19	0.799
		Hallmark Interferon Alpha Response	-0.22	0.791
		Hallmark Oxidative Phosphorylation	-0.48	0.0

High driver count cells from the CRC dataset were highly enriched in several proliferation-associated gene sets: MYC Targets V1 & V2, E2F Targets, G2M Checkpoint, and Mitotic Spindle. CRC driver positive cells (containing either an ERBB2 L755S or a PIK3CA H1047R alteration, or both) were enriched for proliferation-associated gene sets: MYC Targets V1 and P53 Pathway; several signaling pathways, apoptosis, and several immune-related gene sets (Allograft Rejection, and Interferon Alpha & Gamma responses). High driver count cells from the TNBC dataset were enriched for a number of signaling pathways, a proliferative gene set (Mitotic Spindle), Hypoxia and Glycolysis gene sets. The most enriched gene set for the TNBC high drivers group was the WNT Beta Catenin Signaling gene set, which was also significant for the CRC driver positive group. The Wnt/*β*-catenin pathway is heavily implicated in many cancers [24–26], including CRC [27–29] and TNBC [30–32]. In all cases, enrichment for the driver negative/low driver groups was limited and only significant in the case of the Oxidative Phosphorylation gene set enrichment in the low drivers group of the TNBC dataset. These results indicate an enrichment in known, cancer-specific pathways when comparing cells with known drivers or high counts of putative drivers to the remaining cells.

### Putative driver frequencies in TCGA projects

In order to assess the viability of using scRNA-seq variant analysis on other cancer types, we downloaded maf (“mutation annotation format”) files from TCGA for projects containing at least 100 patients and found that most cancer types (20 out of 32) had a median of one or more predicted oncogenic mutations (S1 Table). The top three cancer types for median number of somatic mutations were skin cutaneous melanoma, lung squamous cell carcinoma and lung adenocarcinoma (full results shown in S1 Table).

We also examined the expression of genes with predicted oncogenic alterations and found that, on average, genes with predicted oncogenic alterations are expressed at higher rates than other genes from the same sample, and at higher rates than non-mutated copies of the same gene in other samples from the same project. Statistics summarizing these expression ratios are shown in S1 Table. We also determined percentile statistics for expression of each gene (among all other genes with TPM >1 from the same sample) with at least one predicted oncogenic alteration, and these results are shown in S5 Fig. The mean percentile expression statistic for predicted oncogenic gene expression was 0.708 with a first quartile value of 0.575. These results are generated from processed bulk RNA-seq data and do not account for potential allelic expression imbalances or random dropout events associated with scRNA-seq. However, the previously identified genes with predicted oncogenic alterations have comparatively high expression levels, suggesting that many of the mutations have the potential to be detected by variant calling on the transcriptomics data.

These mutations in TCGA are from WGS or WES data, so they are likely to be present in a large proportion of patients’ tumor cells, tend to be expressed in each patients’ corresponding bulk RNA-seq samples, and may be readily detectable in scRNA-seq data given sufficient gene expression. These results suggest that variant calling on scRNA-seq data may yield cells with putative driver mutations in many cancer types.

### Comparison to WES

We also performed variant calling and annotation on readily available WES data for the TNBC dataset to further validate our findings. Processing and analysis of WES data resulted in fewer putative driver alterations than were found in the scRNA-seq data. However, PT039’s WES data showed two TP53 alterations that appear to be a signature (Y205C and P72R) and that were found in a number of single cells (41 cells for Y205C, 27 cells for P72R, and 24 cells for both).

Some discrepancies may be due to intra-tumor genomic heterogeneity, low sample purity, or a lack of common drivers in the TNBC dataset cells. Both our CNV inference results and those originally presented in [33] suggest that there may be multiple clonotypes for multiple patients, including PT084. Additionally, findings comparing the scRNA-seq-derived inferred CNV to the WES-derived inferred CNV (presented in [33]) suggest that clonotypes present in the scRNA-seq samples may not be present in the WES. Many variants were found in common between each patient’s single cells and their respective WES, and counts of these intersection sizes are shown in S6 Fig. Overall, the WES results provide support for several variants identified in the TNBC scRNA-seq data.

### 10X Genomics NSCLC DTC dataset

10X Genomics scRNA-seq library preparation protocols are widely used and thus present an attractive target for this methodology. However, significantly fewer oncogenic alterations were found in the 10X Genomics NSCLC dataset. Only 16 putative driver alterations, with no more than one per cell, were found in reads corresponding to 13,093 unique cellular barcodes passing filtering in all sequencing runs. We found a median of 96 variants per cell, with almost the entirety being non-coding-sequence variants. Despite the limited efficacy of variant calling on 10X 3’ data, we were able to detect several known recurrent hotspot drivers in single cells, including GTF2I N440S, IRS G1057D, NFE2L2 D77Y, SUZ12 R101*.

## Materials and methods

### Data selection

**Fig 4:**
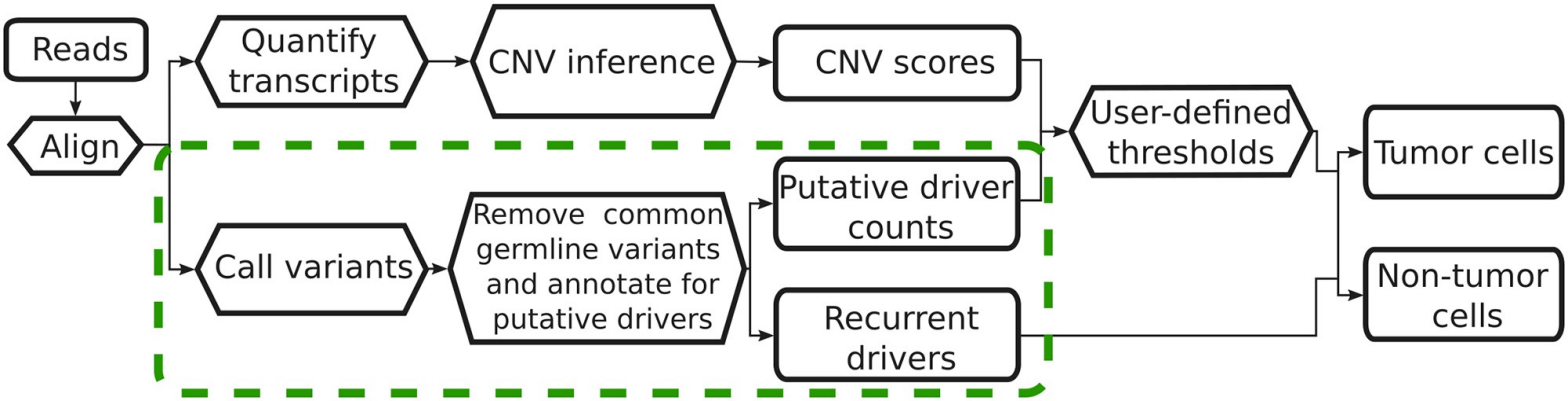
Flowchart depicting a cancer-cell-filtering process. Additional steps proposed in this work, to include variant calling and analysis, are shown by the green dashed rectangle. Solid rectangles indicate inputs and outputs, hexagons indicate processes.

In order to test our approach, we selected two full-length transcript (Smart-seq2) single-cell RNA sequencing datasets from colorectal and triple negative breast cancer tumor samples, respectively, and a 10X Chromium dataset of non-small cell lung cancer for comparison. A flowchart visualizing the general process described in this work is shown in Fig 4. Smart-seq2 generated scRNA-seq data from colorectal cancer (CRC) samples were obtained from the Sequence Read Archive (accession SRP113436) [34]. Triple negative breast cancer (TNBC) scRNA-seq samples, also prepared using the Smart-seq2 protocol, and corresponding whole-exome sequencing (WES) results were obtained from the Gene Expression Omnibus (accession GSE118390) [33]. Matched normal data was not readily available for either dataset. As a basis for comparison, a total of 5,354 healthy pancreas, liver, dermal fibroblast, and neutrophil cells, from samples prepared using Smart-Seq2, were retrieved from [35–37], and [38], respectively. In order to evaluate the potential use of this method with data generated using 10X Chromium (v3.1 chemistry) library preparation, a dataset containing a 20,000 cell mixture of non-small cell lung cancer dissociated tumor cells was obtained from the 10X Genomics website (https://10xgenomics.com/resources/datasets).

### Data processing

#### Quality control

Before performing CNV inference and variant calling, we processed the data to: (1) remove low quality cells; and (2) trim the reads to remove adapters and low-quality basecalls from read ends.

CRC and TNBC cells were initially filtered to remove low quality cells according to the methodology detailed in [33]: cells were selected based on library size, number of expressed genes, and total mRNA. For any of these metrics, cells were excluded if log-transformed values fell below 4 median absolute deviations from the median. In addition to the methodology described in [33], the threshold for the minimum number of expressed genes per cell was raised to a constant value of 1,000, and an additional cell from each dataset was excluded by CopyKAT [10] due to insufficient chromosomal coverage (at one or more chromosomes) to reliably infer CNV. This resulted in 736 viable CRC and 942 viable TNBC cells for analysis.

For variant calling, FASTQs were retrieved using the SRA Toolkit. Trim Galore [39] was used to remove adapters and low-quality basecalls from read ends. For normal tissue cells, 439 cells were excluded by CopyKAT due to insufficient chromosomal coverage, leaving a total of 4,915 normal tissue cells for CNV inference analysis.

#### scRNA-seq processing

After removing low quality cells and trimming reads, we set out to: (1) perform variant calling; (2) remove common germline variants; and (3) annotate the status of the remaining variants as known/predicted oncogenic or not.

Reads were aligned to the most recent hg38 reference genome build (dated March 16, 2020), retrieved from UCSC, using STAR aligner [40]. BCFTools [41] was used to generate genome pileups and to call and filter variants with a minimum quality score of 30. Variants were then annotated using SnpEff [42]. After SnpEff annotation, all common dbSNP variants (retrieved from https://ftp.ncbi.nih.gov/snp/organisms/human_9606_b151_GRCh38p7/VCF/00-common_all.vcf.gz) were removed in order to avoid using common germline variants as evidence towards the cancer status of cells. Coding sequence variants were then annotated using the OncoKB API [13] to obtain any known or predicted oncogenicity for the variant. The OncoKB API use requires an API token, obtainable at https://oncokb.org/apiAccess. GNU parallel [43] was used for the parallelization of a number of steps described here. A complete pipeline producing these results is available in the project code repository.

#### Coverage quantification

The next step in our approach is to determine whether individual SNVs are truly absent in a sample or there is not enough evidence to make the call. The reason for this is that normal variations in gene expression, random dropout, capture inefficiencies, and other sources of noise make it impossible to determine that SNVs are not present in a sample unless the sample has sufficient coverage at the corresponding genomic position. To this end, a Python script was used to determine whether it is likely to effectively call an SNV at any given genomic position, based on a required minimum read depth of 5 at the respective position. This minimum depth is only used to infer the absence of a variant (for Fig 2), as no assumptions about genome coverage can be made for transcriptomics data. For calls about variant presence, a minimum quality score of 30 was used, as described in the prior section.

After alignment and annotation, all putative driver alterations were extracted from annotated VCF files. For each residue-level putative driver alteration, the genomic positions corresponding to the residue were then looked up using the most recent hg38 GTF file (retrieved from UCSC, dated December 12, 2021) and stored. Then, for each sample, genome pileups were generated from sorted BAMs using BCFTools and piped into a Python script, which measured coverage at all relevant genomic positions. In analysis, this coverage data was used to determine whether a putative driver alteration was likely absent or if there was insufficient coverage to call either way.

### CNV inference

Our approach for identifying cancer cells combines variant calling of known/putative drivers with CNV inference. CNV inference was performed using CopyKAT [10] with default parameters and a window size of 100. The TPM matrix originally produced in [33] (available under GSE118390) was used as input for CopyKAT. For CRC and normal tissue cells, transcript quantification was performed using htseq-count from HTSeq [44] in intersection-strict mode, converted to TPM, and then CNV was inferred using CopyKAT.

### Gene set enrichment analysis

In the absence of a gold standard detailing what cells in a dataset are actual cancer cells, we performed gene set enrichment analysis to determine whether our approach yielded cells with expression profiles enriched in cancer pathways. Raw counts matrices, generated using htseq-count [44], were normalized using DESeq2 [45]. DESeq2-normalized counts were then used with the Broad Institute GSEA implementation [22] and groups of samples were analyzed for enrichment in MSigDB hallmark gene sets [23].

### WES data processing

In order to determine what proportion of mutation calls in single-cell RNA datasets were also present in bulk RNA sequencing data and vice versa, we analyzed whole-exome sequencing (WES) data available in the triple negative breast cancer (TNBC) dataset. WES samples from TNBC patients used for validation were initially trimmed to remove adapters and low-quality basecalls using Trim Galore [39]. Reads were aligned to the most recent hg38 reference genome build (dated March 16, 2020) using BWA [46] and sorted using SAMTools [41]. PCR duplicates were removed using GATK’s MarkDuplicates [47]. BCFTools was used for generating genome pileups, variant calling, and variant filtering. SnpEff and the OncoKB API were used for annotating final variant calls.

### 10X data processing

Although we hypothesized that our approach would only work reliably in full-length transcript data, we wanted to determine its applicability on data generated with the 10X Chromium platform. The 10X NSCLC 20k mixture dataset was initially processed using Cell Ranger [48] from 10X Genomics. For each cell, reads corresponding to each unique cell barcode were extracted from all sequencing runs and sorted. Variant calling proceeded similarly to the TNBC dataset processing: BCFTools was used to generate pileups and call variants, and variants were annotated using SnpEff.

## Discussion and conclusion

This work demonstrates the viability of calling the presence and absence of oncogenic variants in scRNA-seq data. Variants can be readily identified in scRNA-seq data generated by the Smart-seq2 protocol, and these variants may be used to increase confidence when filtering data to exclude non-tumor cells. This approach is particularly useful when cells lack major structural alterations (Fig 3 and S4 Fig). Although CNV inference will not likely yield false positives when selecting cells with high inferred CNV, it may result in false negatives if used to exclude cells with low inferred CNV.

Aside from CNV inference, other methodologies for cancer cell filtering may produce both false negatives and false positives. Cluster-based filtering may require arbitrary boundary decisions that may include or exclude cells incorrectly, and clonotypes represented by small numbers of cells may not produce distinct clusters. Marker gene expression may effectively allow for the exclusion of certain cell types, particularly immune and endothelial cells, but will not help to discriminate between tumor and normal epithelial or stromal cells (e.g., EMT). Additionally, tumor-macrophage fusion cells [49, 50] may be incorrectly called immune cells when using marker-based exclusion methods.

The results presented here illustrate the need for augmenting CNV inference with additional evidence to confidently call cancer cells in scRNA-seq datasets. We found that many cells with low inferred CNV harbored high numbers of putative oncogenic alterations, as shown in Fig 3, and that 458 low-inferred-CNV cells from the CRC dataset are predicted to harbor recurrent hotspot driver alterations (Fig 2). Additionally, there is not always a correlation between inferred CNV and the number of putative driver alterations identified (Table 1). If cell filtering on this data were based on CNV inference alone, then many likely-cancer cells would be excluded. This phenomenon is particularly pronounced for the vast majority of CRC cells, which had comparatively limited inferred CNV scores but had high numbers of putative oncogenic alterations or recurrent driver mutations. We suspect that a number of these cells are genuinely cancer cells with limited CNV. Major structural alterations are not required for tumorigenesis and disease progression, and tumors with minimal structural alterations but comparatively high mutation burden have been observed in prior work [11, 51, 52].

The approach presented here may yield both false negatives and false positives. As shown in Fig 2 and S1 Fig, coverage is highly variable and coverage at residues of interest must be explicitly examined to predict the absence of a variant. Given coverage issues and the potential for allele-specific expression, it may be difficult to definitively say that a cell lacks a given variant or that a cell is definitively normal because no driver alterations were detected. However, cancer positive status for a cell is likely if driver alterations are detected, especially when they are recurrent drivers (as is the case for the CRC dataset) or when they are detected in high numbers. These metrics should be used in conjunction with other methods, as there is also some potential for false positives. Many of the confounding technical factors that are problematic for all scRNA-seq analyses (including expression marker-based filtering and CNV inference) may also cause issues for variant calling and analysis. Doublets, where multiple cells are processed as a single sample, may confound analyses when cells with differing phenotypes are combined. Additionally, ambient RNA can be internalized through endocytosis by cells in the tumor microenvironment [53, 54] and may cause false positives when performing variant calling. There is also some possibility that transcripts from cancer cells may be found in normal cells through macrophage phagocytosis of cancer cells or through the endocytosis of cancer-derived exosomes or free mRNA. Interestingly, we found small numbers of putative immune cells (cells expressing common immune markers) containing either elevated putative driver counts or, in the case of the CRC dataset, known drivers (as indicated by the immune pathway enrichment in Table 2). These cells frequently contained high numbers of reads (> 50) supporting each call, and this may be the result of doublets, ambient RNA, or natural biological phenomena (macrophage phagocytosis of cancer cells, exosomes, or extracellular material; or dendritic cell environmental sampling, for example).

Our work provides a way to bolster cell filtering with additional evidence. In isolation, this method may not be sufficient to identify cancer cells in a highly reliable way: normal expression variation and other sources of noise suggest that oncogenic alterations will not always be consistently present in the scRNA-seq data for cancer cells. Nor do we suggest that the presented method, or any other methodology, should be used in isolation when attempting to filter cancer cells. As previously mentioned, variant analysis alone may result in both false positives and false negatives; CNV inference may result in false negatives, as demonstrated by the CRC dataset in this work; and expression-based methods utilizing epithelial or stromal markers for inclusion are non-definitive. Our work aims to provide an orthogonal methodology that may complement existing filtering techniques. We showed that this approach effectively augments calls made using alternate methods and is especially interesting in the context of CNV inference data. The added dimension provided by putative driver alteration counts can effectively separate cells that are indistinguishable by CNV alone.

Unfortunately, there are a handful of current issues preventing the use of this method with 10X-generated data. The 10X Genomics 3’ protocol generates reads that only cover a short region of the 3’ end of each transcript captured. This poses a serious limitation for the identification of oncogenic coding sequence alterations, which are the focus of this work. Most of the target genes examined here have a 3’ untranslated region longer than the short reads typically sequenced in scRNA-seq experiments. However, there does seem to be potential for future studies in this area, as recurrent hotspot alterations were identified in a very small proportion of cells in 10X Genomics data.

The presence of putative driver alterations in some normal cells is an interesting finding. While there may be comparatively less confidence in scRNA-seq variant calling compared to WES or WGS, we suspect that at least some of the variant calls represent genuine somatic mosaicism. However, somatic mosaicism is unlikely to be a confounding factor when examining putative driver alteration counts in cancer datasets. The putative driver alterations counts distribution for the normal cells (shown in S3 Fig) illustrates that only limited numbers of putative driver alterations can be found in cells with a known non-cancer status. The high numbers of cells from cancer datasets that have putative driver alteration counts greater than the 99th percentile of the 5,354 normal cells (shown in S7 Fig) suggests that the normal counts distribution effectively describes the degree to which somatic mosaicism may confound our analyses. The putative drivers used as evidence for the cancer cell status are backed by data that suggests detriment to normal cellular functions (i.e., internal homeostasis) in the presence of these genetic alterations.

There appears to be only a limited body of work quantifying the expected rates of short variant single-cell somatic mosaicism. Lodato et al. found varying rates of single-cell somatic SNV accumulation in neurons (up to ≈ 40 per year) and that single-cell SNV counts strongly correlated with age [55]. Large scale single-cell somatic mosaicism has also been found in neurons: McConnell et al. found that 13−41% of frontal cortex neurons harbor megabase-scale *de novo* CNVs [56]. Expected short variant single-cell somatic mosaicism rates for other normal tissues do not appear to be well characterized. Widespread single-cell short variant somatic mosaicism might be expected based solely on DNA replication error rates [57, 58], but there does not appear to be extensive experimental evidence to support this. These results indicate the need for additional experimental investigation into the normal cell space to confirm whether the variants found in data from healthy individuals (Fig 3 and S3 Fig) are due to artifacts during single cell collection (e.g., doublets that are a common problem during fluorescence-activated cell sorting), library preparation, or whether they represent true genetic alterations with potential unexplored biological implication.

Connected to this observation, we also note that the very definition of what constitutes a cancer cell is ultimately somewhat fraught. Cancer is generally defined as a set of behavioral traits (“hallmarks”) acquired by a cell over a period of time that lead to–among several other traits–uncontrolled proliferation [59, 60]. Genomic and epigenomic events and the microenvironment are normally underpinning the acquisition of these hallmarks, but genotype-phenotype relationships in cancer are usually not straightforward or one-to-one [61]. In addition, tissues adjacent to cancer cells have been shown to contain abnormal genetic alterations [62], and their transcriptomics profiles are often in an intermediate state between that of normal and tumor cells [63]. These observations point to the fact that computational approaches can effectively enrich for true cancer cells, but a clear-cut distinction between “true” cancer cells and pre-neoplastic cells is often extremely difficult to make.

Certain cancer types may be more amenable to our proposed methodology than others, and cancers with high mutation burden and low CNA burden may be the most viable targets. A number of cancer types, such as skin, lung, certain lymphomas, and various squamous cell carcinomas have comparatively high mutation burdens, as shown in [64] and confirmed in our own analysis (S1 Table) and may be most likely to harbor large numbers of putative driver alterations. Our analysis of putative driver mutations in TCGA projects (S1 Table) suggests that, for a number of cancers, it should frequently be possible to find common recurrent driver mutations, like those found in the CRC dataset in this work. It should be noted that normal cells may show elevated putative driver counts for mutagen-induced cancers, like skin and lung cancers, as normal cells exposed to mutagens may accumulate putative drivers at higher rates than might be expected for normal cells that are not exposed to mutagens. Additionally, characteristic, high-frequency mutations may be identified in cells to increase confidence in cancer status. These characteristic mutations may be limited to the context of specific patients, like the TP53 Y205C and P72R mutations in PT039’s cells, but may more broadly be found in certain cancer types, as in the case of the ERBB2 L755S and PIK3CA H1047R alterations in the CRC dataset, or the KRAS G12x mutation, which can be found in 64.9% of pancreatic adenocarcinoma samples aggregated on CBioPortal [65].

In summary, this work presents a viable methodology for augmenting the cancer-cell-filtering process in scRNA-seq datasets. We found that known, recurrent driver mutations could be detected in a number of cells and provide meaningful evidence towards the cancer status of the cell. We also found that the counts of these putative driver alterations differed significantly from what can be found in normal (i.e., non-tumor) cells. Furthermore, there does not appear to be a consistent correlation between inferred CNV and putative driver alteration counts. Our method then provides evidence supporting the inclusion in downstream analyses of low-inferred-CNV cells that might otherwise be excluded and can strengthen the confidence in calls made on high-inferred-CNV cells.

## Supporting information

S1 Fig Heatmap indicating alteration status for 942 cells for the top 25 most frequent oncogenic, predicted oncogenic, and likely oncogenic alterations in the CRC dataset. Alterations are annotated using OncoKB. Absence of an alteration is noted when a cell has a read depth of at least 5 for all bases corresponding to the residue. For residues without an oncogenic alteration and with read depths less than 5 for all corresponding bases, the presence or absence of an alteration is not characterized (“Insufficient coverage”).
(TIF)

S2 Fig Counts of splice site variants for CRC, TNBC, and normal dataset cells. Significant differences were found between CRC and normal cell distributions (Mann-Whitney U; *p* = 9.75 ⋅ 10^−230^), and between TNBC and normal cell distributions (Mann-Whitney U; *p* = 0.0016), however effect size for TNBC versus normal was negligible (Cliff’s delta; *δ* = 0.064).
(TIF)

S3 Fig Boxplots showing putative driver alteration counts distributions for all normal tissue cells combined and TNBC and CRC dataset cells. (TIF)

S4 Fig Boxplots showing mean absolute CNV scores for all normal tissue cells combined and TNBC and CRC dataset cells. (TIF)

S5 Fig Box plot showing the expression percentile statistics for genes with predicted oncogenic alterations in bulk RNA-seq samples corresponding to patients with predicted oncogenic somatic mutations (identified in WGS or WES). Figure was generated using genomic and transcriptomics data retrieved from TCGA.
(TIF)

S6 Fig Heatmaps showing the intersection sizes of genomic variants found in single cells and each patient’s respective WES samples. (TIF)

S7 Fig Histogram showing the distribution of putative driver alteration counts in 5,354 normal cells. Dashed line indicates 99th percentile.
(TIF)

S1 Table Median somatic mutation and predicted oncogenic mutation counts for patients from TCGA projects. All somatic mutations are annotated using OncoKB to determine predicted oncogenicity. Projects are ordered by median number of mutations. Fourth column shows the mean of expression ratios describing the expression of oncogenic genes divided by the median expression within the same sample. Fifth column shows the mean of expression ratios describing the expression of genes with predicted oncogenic mutations divided by the median of expression for non-mutated versions of those same genes in other samples from the same project.
(PDF)
