# Supplementary figures and images for: Variant calling enhances the identification of cancer cells in single-cell RNA sequencing data
Source: PLoS Comput Biol. 2022 Oct 3;18(10):e1010576. doi: 10.1371/journal.pcbi.1010576 (PMC9560611; doi:10.1371/journal.pcbi.1010576)

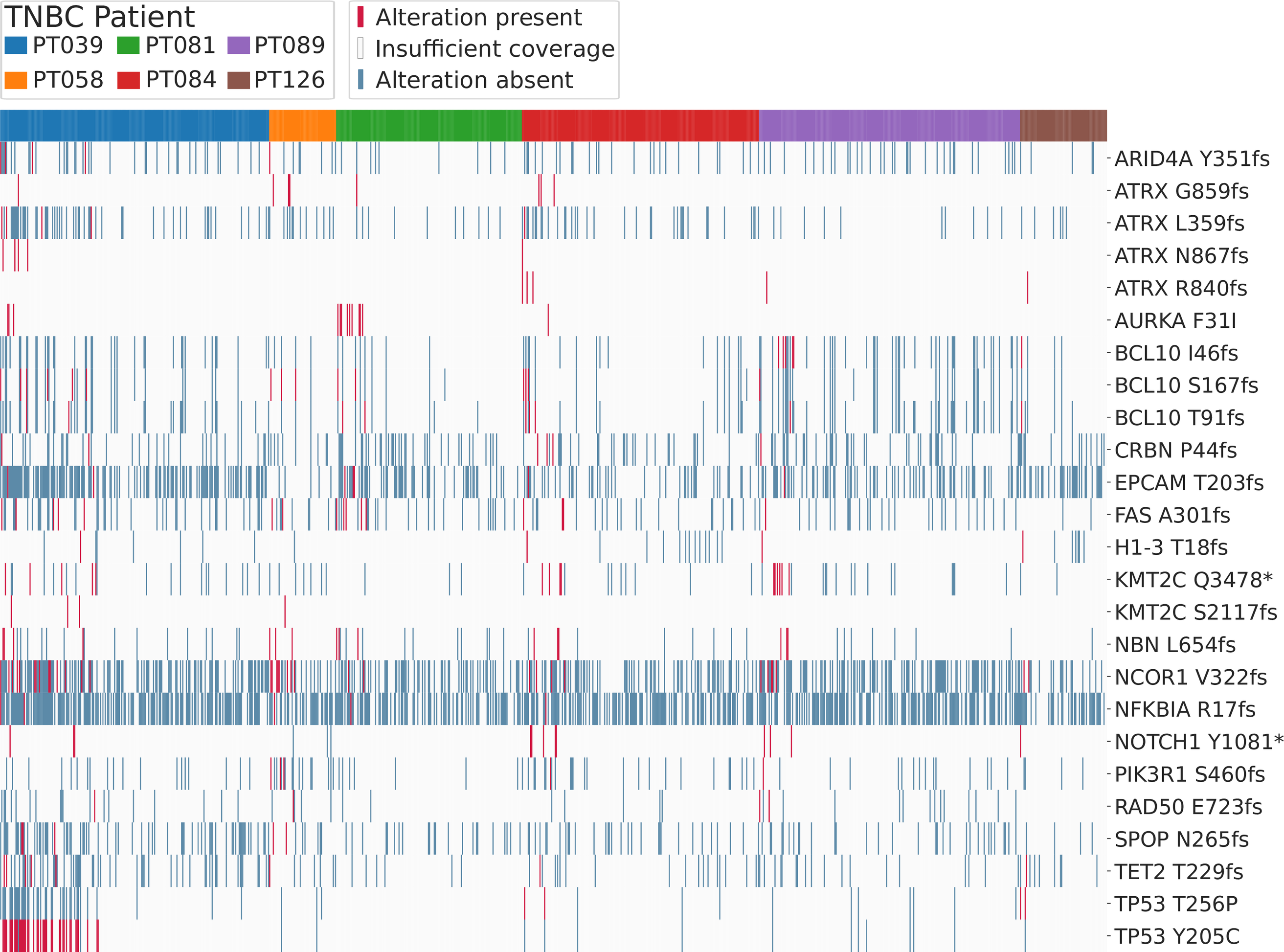

Supplement: S1 Fig — Alterations are annotated using OncoKB. Absence of an alteration is noted when a cell has a read depth of at least 5 for all bases corresponding to the residue. For residues without an oncogenic alteration and with read depths less than 5 for all corresponding bases, the presence or absence of an alteration is not characterized (“Insufficient coverage”). (TIF) [file pcbi.1010576.s001.tif]

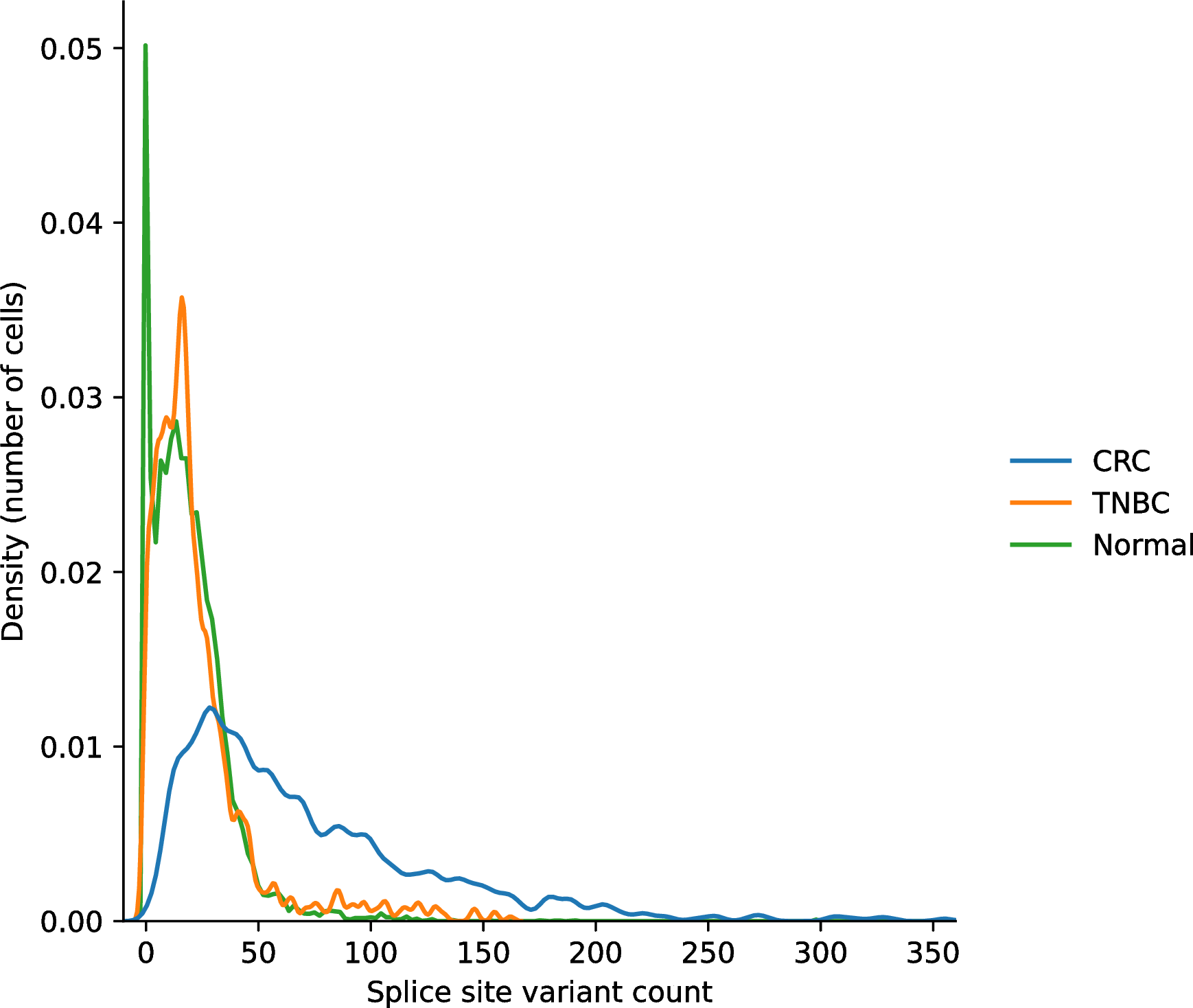

Supplement: S2 Fig — Significant differences were found between CRC and normal cell distributions (Mann-Whitney U; p = 9.75 ⋅ 10−230), and between TNBC and normal cell distributions (Mann-Whitney U; p = 0.0016), however effect size for TNBC versus normal was negligible (Cliff’s delta; δ = 0.064). (TIF) [file pcbi.1010576.s002.tif]

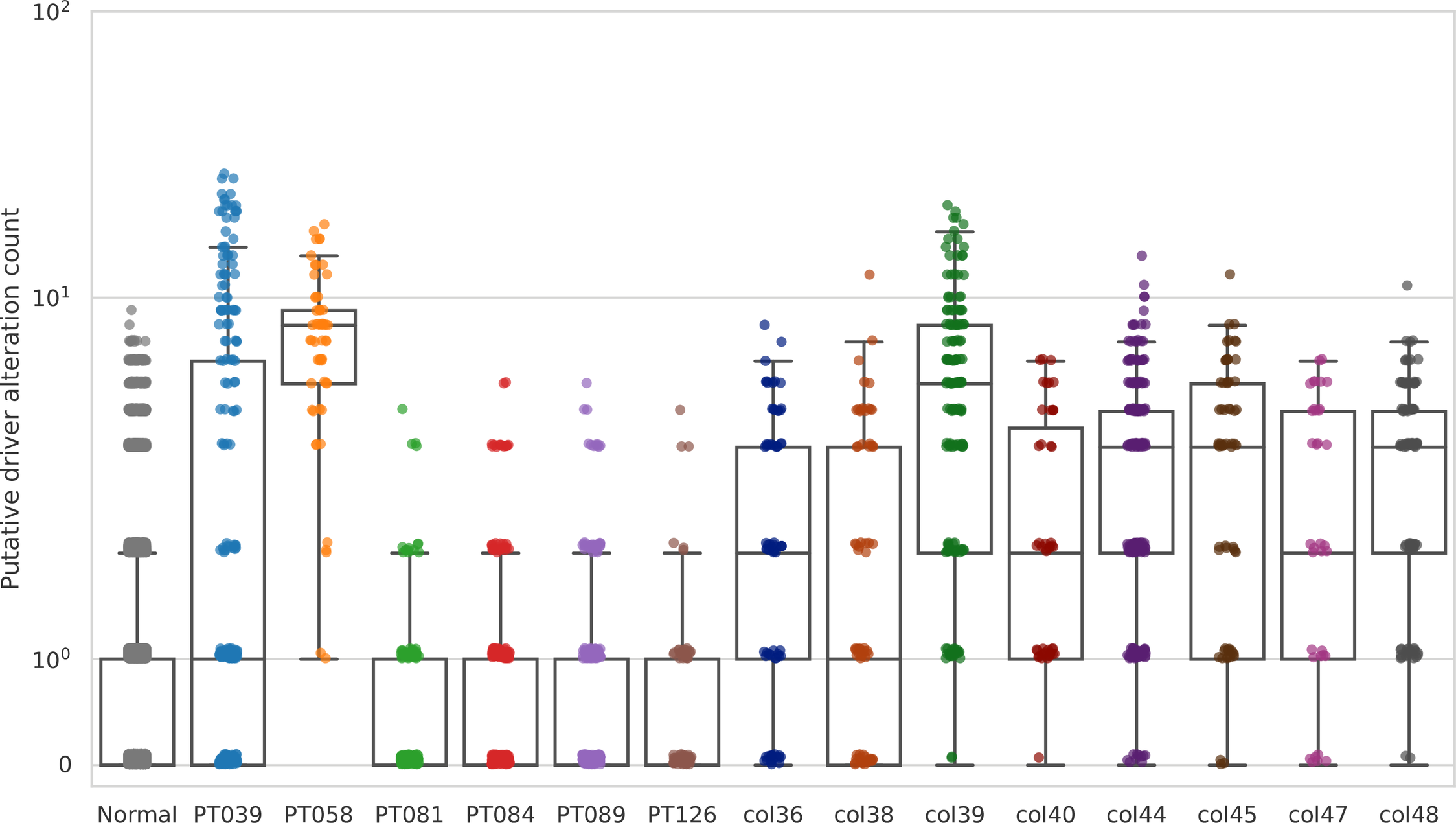

Supplement: S3 Fig — (TIF) [file pcbi.1010576.s003.tif]

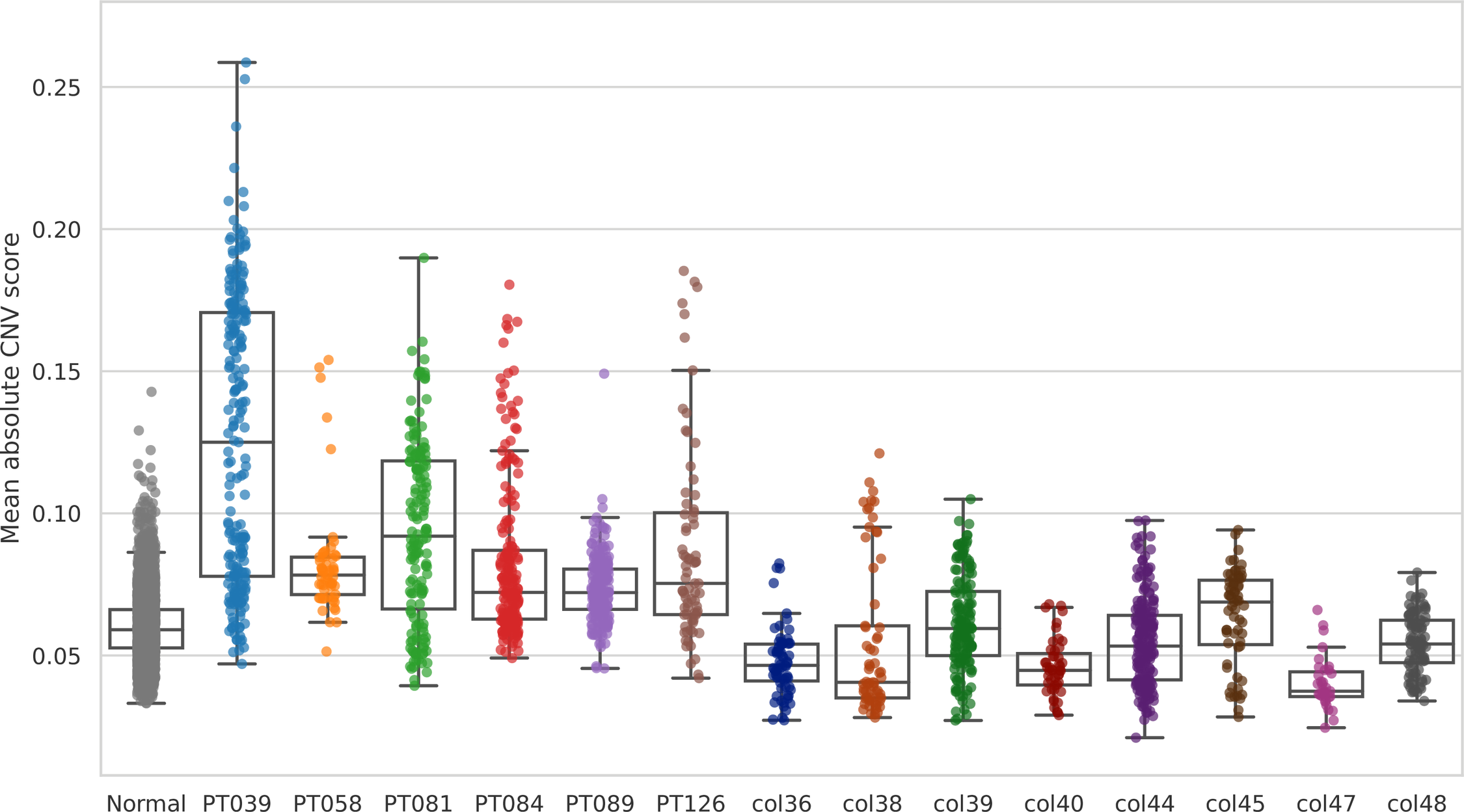

Supplement: S4 Fig — (TIF) [file pcbi.1010576.s004.tif]

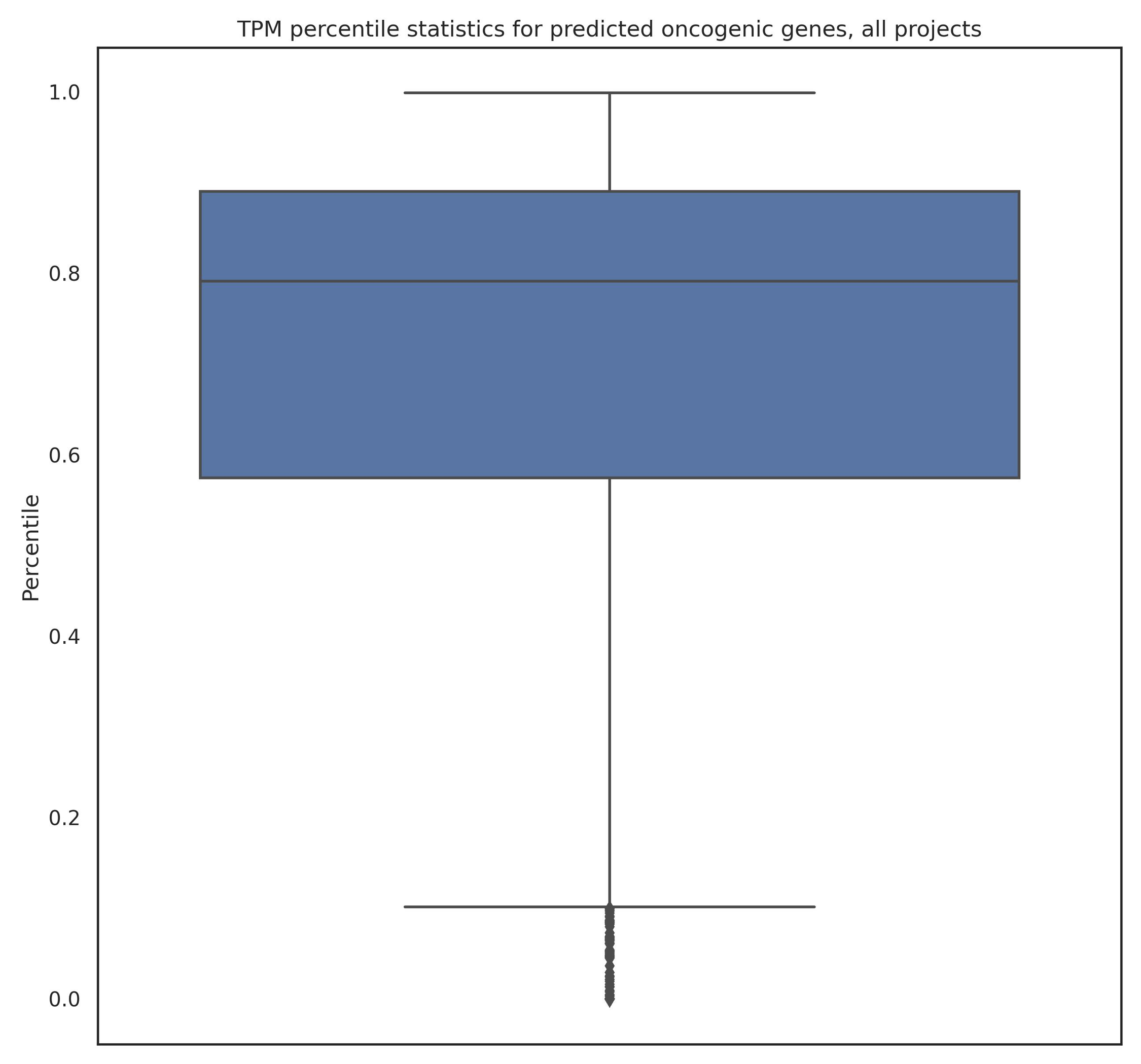

Supplement: S5 Fig — Figure was generated using genomic and transcriptomics data retrieved from TCGA. (TIF) [file pcbi.1010576.s005.tif]

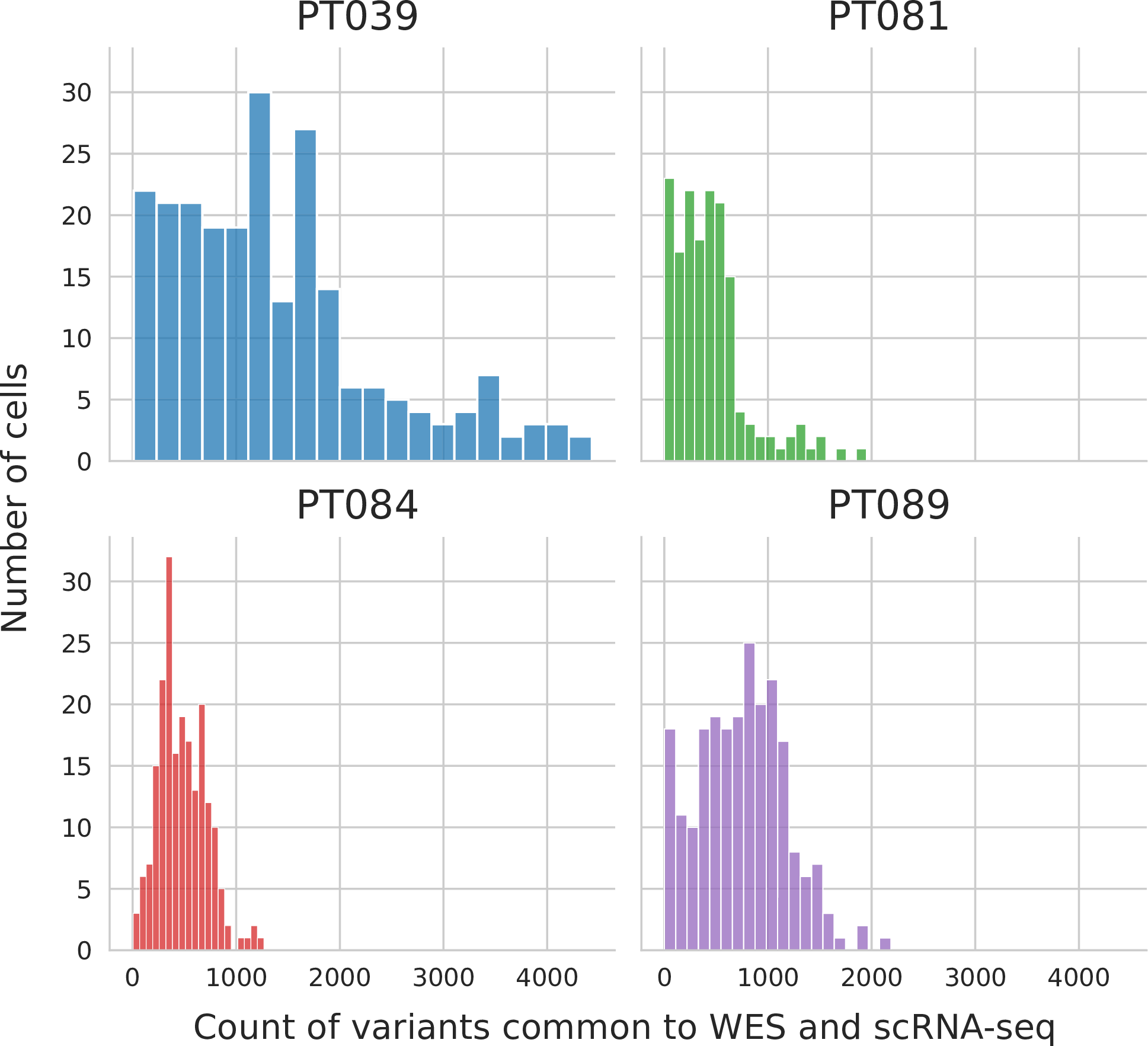

Supplement: S6 Fig — (TIF) [file pcbi.1010576.s006.tif]

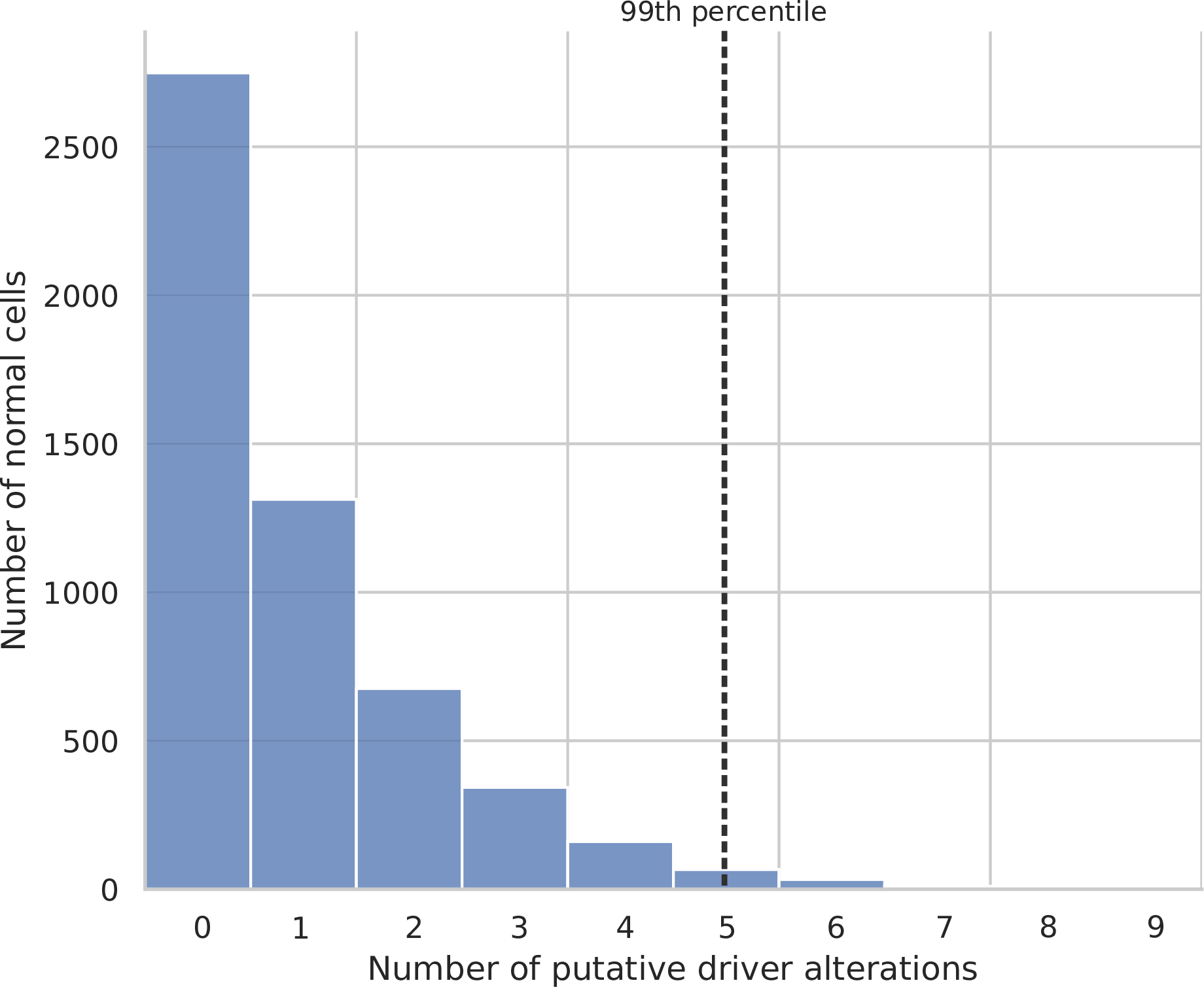

Supplement: S7 Fig — Dashed line indicates 99th percentile. (TIF) [file pcbi.1010576.s007.tif]
