# Supplementary material for: Variant calling enhances the identification of cancer cells in single-cell RNA sequencing data
Source: PLoS Comput Biol. 2022 Oct 3;18(10):e1010576. doi: 10.1371/journal.pcbi.1010576 (PMC9560611; doi:10.1371/journal.pcbi.1010576)
Supplement: S1 Table — All somatic mutations are annotated using OncoKB to determine predicted oncogenicity. Projects are ordered by median number of mutations. Fourth column shows the mean of expression ratios describing the expression of oncogenic genes divided by the median expression within the same sample. Fifth column shows the mean of expression ratios describing the expression of genes with predicted oncogenic mutations divided by the median of expression for non-mutated versions of those same genes in other samples from the same project. (PDF) [file pcbi.1010576.s008.pdf]

Project	Median number of mutations	Median number of predicted oncogenic mutations	Mean expression ratio: mutated genes vs. all other genes (within sample)	Mean expression ratio: mutated vs. non-mutated (same gene, across samples)
TCGA-SKCM	235.5	1.0	4.4581	1.2425
TCGA-LUSC	171.0	1.0	5.4145	2.1433
TCGA-LUAD	145.0	1.0	3.8185	1.2653
TCGA-BLCA	128.5	1.0	4.0413	1.2947
TCGA-DLBC	101.0	1.5	8.6160	0.9516
TCGA-COAD	82.0	2.0	2.3204	1.0383
TCGA-STAD	82.0	1.0	3.7006	1.1058
TCGA-ESCA	78.5	1.0	5.5755	2.3456
TCGA-HNSC	71.0	1.0	3.3772	2.0338
TCGA-UCEC	56.0	2.0	2.4052	1.1217
TCGA-CESC	63.0	1.0	1.8355	1.1178
TCGA-READ	66.0	2.0	2.4815	3.1147
TCGA-LIHC	59.0	0.0	3.8100	1.1736
TCGA-OV	49.0	1.0	10.125	4.5262
TCGA-UCS	37.0	2.0	4.0500	1.1461
TCGA-GBM	39.0	1.0	5.2225	1.2357
TCGA-KIRP	40.0	0.0	3.3697	1.2626
TCGA-KIRC	35.5	0.0	1.3654	0.9224
TCGA-BRCA	28.0	1.0	2.1808	1.1667
TCGA-SARC	28.0	0.0	3.7450	1.5817
TCGA-PAAD	25.0	1.0	2.9296	1.3135
TCGA-CHOL	24.0	1.0	8.7314	1.3068
TCGA-ACC	17.0	0.0	8.3805	1.1639
TCGA-LGG	19.0	2.0	4.4989	0.8806
TCGA-PRAD	18.0	0.0	4.1470	0.9803
TCGA-MESO	18.0	0.0	3.6020	0.7598
TCGA-KICH	11.0	0.0	1.5548	1.2690
TCGA-THYM	7.5	1.0	1.0929	2.0145
TCGA-TGCT	10.0	0.0	7.2599	1.1569
TCGA-THCA	6.0	0.0	3.4057	1.0529
TCGA-PCPG	6.0	0.0	17.795	1.9755
TCGA-LAML	3.0	0.0	12.630	1.0162
